# A Rare Case of Human Herpesvirus 6 Meningitis in an Immunocompetent Adult Presenting With Recurrent Encephalopathy: Could Prior Head Trauma or an Incidental Cerebral Cavernous Malformation Be a Predisposing Factor?

**DOI:** 10.7759/cureus.87511

**Published:** 2025-07-08

**Authors:** Zhuo Luan

**Affiliations:** 1 Neurology, Texas Tech University Health Sciences Center El Paso, EL Paso, USA

**Keywords:** cerebral cavernous malformation, human herpesvirus-6 (hhv-6), immunocompetent adult, trauma, viral meningitis

## Abstract

Human herpesvirus 6 (HHV-6) meningitis/encephalitis is typically seen in immunocompromised patients, with rare reports in immunocompetent adults. We present the case of a 52-year-old immunocompetent man with recurrent acute encephalopathy and incidental cerebral cavernous malformation (CCM), alongside a remote history of traumatic brain injury (TBI). Cerebrospinal fluid (CSF) analysis and multiplex polymerase chain reaction (PCR) test confirmed HHV-6 infection, with neuroimaging showing leptomeningeal enhancement. This case suggests that structural brain abnormalities such as CCM and prior TBI may cause chronic blood-brain barrier (BBB) disruption, facilitating HHV-6 neuroinvasion even in the absence of immunosuppression. Awareness of such predisposing factors is crucial for the timely diagnosis and treatment of HHV-6 meningitis/encephalitis in immunocompetent individuals. Further research is needed to clarify the pathophysiological link between BBB dysfunction and HHV-6 central nervous system (CNS) infection.

## Introduction

Human herpesvirus 6 (HHV-6) is a beta (β)-herpesvirus that typically establishes lifelong latency after primary infection, which occurs in the majority of individuals during early childhood. While often asymptomatic or associated with mild febrile illness in children (such as roseola infantum), HHV-6 can reactivate under conditions of immunosuppression and cause a range of serious complications. In immunocompromised patients, including those with HIV or recipients of solid organ or hematopoietic stem cell transplants, HHV-6 reactivation is a well-recognized cause of severe central nervous system (CNS) pathology, such as limbic encephalitis, meningoencephalitis, and cognitive dysfunction [1]. In contrast, HHV-6 meningitis/encephalitis in immunocompetent individuals is rare, poorly understood, and likely under-recognized.

The mechanisms by which HHV-6 invades the CNS are not fully elucidated. One of the mechanisms of the proposed route of entry is through disruption of the blood-brain barrier (BBB), a highly specialized endothelial interface that regulates molecular and cellular traffic between the peripheral circulation and the CNS [2]. Under normal physiologic conditions, the BBB restricts viral penetration and helps maintain the immune integrity of the CNS. However, local inflammatory responses, direct endothelial damage, or structural abnormalities can compromise BBB integrity and allow viral access to the brain parenchyma [2]. Emerging evidence suggests that structural brain lesions such as cerebral cavernous malformations (CCMs) and prior traumatic brain injury (TBI) may contribute to chronic BBB dysfunction. CCMs are clusters of dilated, thin-walled capillaries that lack intervening neural tissue and are often associated with microhemorrhages, hemosiderin deposition, gliosis, and localized inflammation, all of which may impair BBB function [3]. Similarly, TBI is known to result in both acute and chronic disruption of the BBB through mechanisms including mechanical endothelial damage, oxidative stress, and tight junction dysregulation. These effects can persist for years after the initial insult, creating a sustained environment of vascular permeability and increased susceptibility to neuroinflammatory insults [4]. Whether such chronic structural vulnerabilities in the BBB could permit viral pathogens such as HHV-6 to penetrate the CNS in the absence of systemic immunosuppression remains an open question. To our knowledge, no case reports have examined this possible link.

Here, we describe a rare case of HHV-6 meningitis in an immunocompetent middle-aged man who presented with recurrent episodes of acute encephalopathy. Neuroimaging revealed an incidental CCM, and his history was notable for a TBI. This unique clinical context raises the possibility that longstanding BBB dysfunction, due to either the CCM, the prior TBI, or both, may have created a permissive environment for HHV-6 reactivation or direct invasion of the CNS. In the absence of traditional immunosuppressive risk factors, this case underscores the need to consider alternative predisposing mechanisms, such as structural or acquired BBB impairment, in the pathogenesis of HHV-6 meningitis/encephalitis in otherwise healthy individuals. Further investigation is warranted to explore this potential association and its implications for diagnosis, risk stratification, and management.

## Case presentation

A 52-year-old man with no significant comorbidities presented to the emergency department with a four-day history of headache, followed by one day of acute confusion, agitation, and disorganized speech without seizure-like activities. Despite the administration of midazolam and ketamine for sedation, his agitation remained uncontrolled. He was subsequently intubated for severe aggression and agitation and admitted to the medical intensive care unit. Three weeks prior, the patient had experienced a similar but transient episode of confusion, which resolved within nine hours. At that time, he underwent an EEG and brain MRI at another hospital, both of which were reportedly unremarkable.

He had a history of TBI in 2016 following a mechanical fall, without documented intracranial injury, but he reported chronic post-traumatic migraine-type headaches since then. According to the family, the patient did not use alcohol, tobacco, or illicit drugs. Notably, a coworker who worked closely with the patient was diagnosed with aseptic meningitis of unknown etiology after presenting with confusion three months earlier. A few weeks after his colleague’s diagnosis, the patient experienced transient neurological symptoms, specifically speech difficulty that lasted a few hours and resolved spontaneously. He did not seek medical attention at the time, so the underlying cause remains unknown, though a possible association with HHV-6 cannot be excluded.

**Figure 1:**
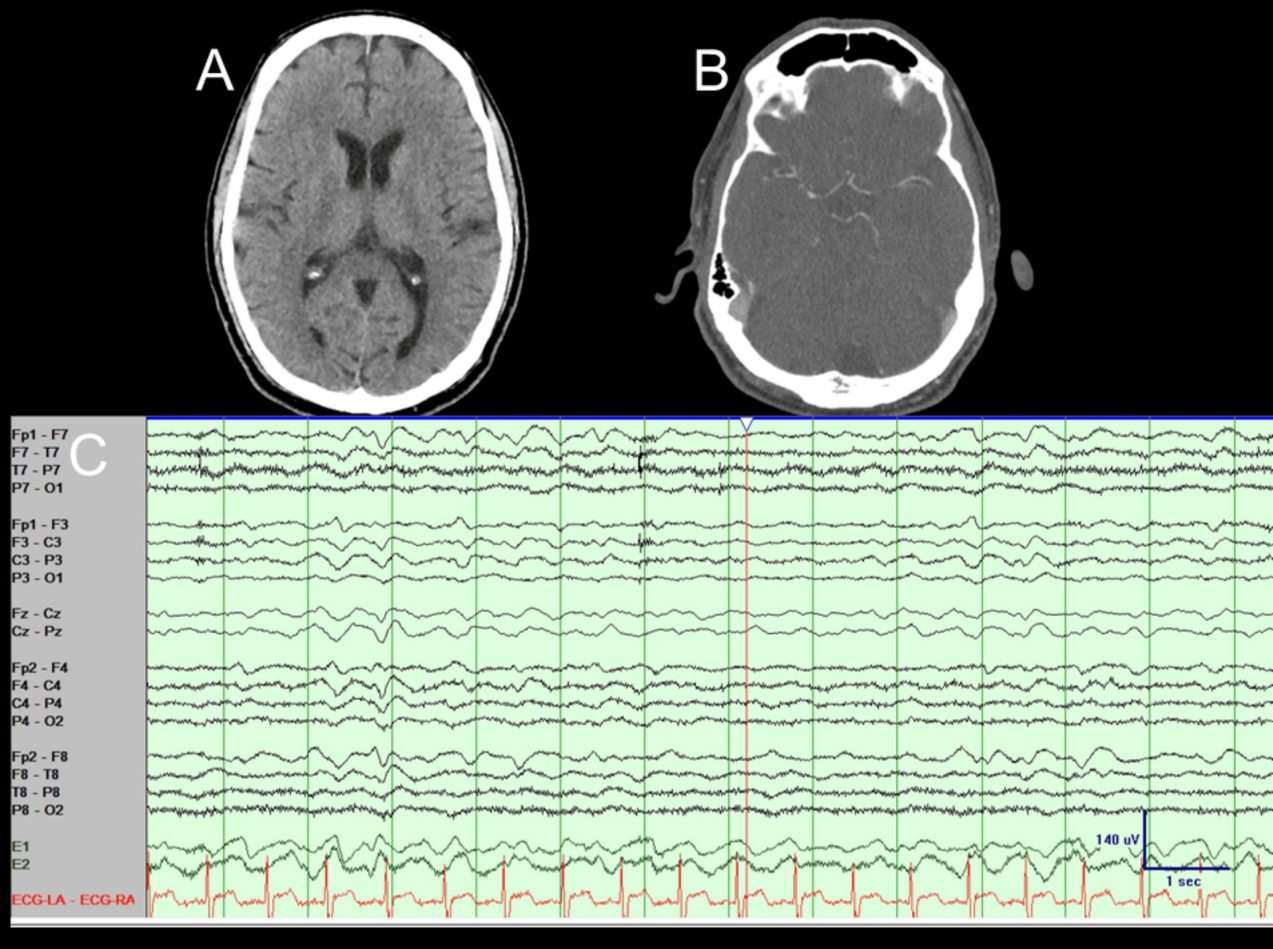
Initial diagnostic workup including CT and CTA of the head and EEG CT and CTA of the head were negative (A and B). The EEG (C) showed diffuse slowing background lacking normal awaking or sleep organization, without discernible posterior dominant rhythm and non-epileptiform abnormalities or electro-clinical seizures, which is suggestive of moderate diffuse encephalopathy. CTA: CT angiogram; EEG: electroencephalogram

Initial CT and CT angiography (CTA) of the head were negative (Figures 1A, 1B), and the initial EEG demonstrated moderate diffuse encephalopathy without epileptiform discharges or seizures (Figure 1C). Lumbar puncture revealed clear, colorless cerebrospinal fluid (CSF) with a white blood cell count of 26/mm³ (99% lymphocytes), protein values of 221 mg/dL, and glucose values of 47 mg/dL. The BioFire meningitis/encephalitis panel (a multiplex nested polymerase chain reaction (PCR) assay with a specificity of approximately 97%) (BioFire Diagnostics, LLC, Salt Lake City, UT)) was performed to test for multiple pathogens, including *Escherichia coli* K1, *Haemophilus influenzae*, *Listeria monocytogenes*, *Neisseria meningitidis*, *Streptococcus agalactiae*, *Streptococcus pneumoniae*, cytomegalovirus (CMV), *Cryptococcus neoformans*/*Cryptococcus *gattii, enterovirus, herpes simplex virus types 1 and 2 (HSV-1 and HSV-2), human parechovirus, varicella-zoster virus (VZV), and HHV-6 [5]. HHV-6 was the only pathogen detected (Table 1).

**Table 1:** Cerebrospinal fluid (CSF) analysis Total nucleated cells, protein, and lymphocyte percentage in the CSF were elevated. Meningitis panel detected HHV6. RBC: red blood cell; SEG: segmented neutrophils; HHV-6: human herpesvirus 6

CSF	Value	Reference range
Total nucleated cells	26	0-5/uL
RBC	1	0-5/uL
Glucose	47	40-70mg/dL
Protein	221	12-60mg/dL
SEG CSF%	1%	0%-2%
Lymph CSF%	99%	63%-99%
Macrophage CSF %	0%	3%-37%
Meningitis panel	HHV-6 detected	N/A
CSF culture	Negative	N/A

Additional laboratory studies were ordered to rule out other infectious etiologies, including HIV, syphilis, tuberculosis, cryptococcosis, histoplasmosis, and coccidioidomycosis, all of which returned negative (Table 2). HHV-6 remained the only identified pathogen.

**Table 2:** Serological and urine lab work The patient tested negative for HIV, syphilis, tuberculosis, cryptococcosis, histoplasmosis, and coccidioidomycosis and was negative for a urine drug test.

Serum	Value	Reference range
Ammonia	<8.7	9-30 umol/L
Rapid plasma reagin	Non-reactive	Non-reactive
Crypto antigen	Negative	Negative
Coccidioides antibodies	<1:2	<1:2
QuantiFERON®-TB Gold	Negative	Negative
HIV ½ enzyme immunoassay	Negative	Negative
Blood culture	Negative	Negative
Urine	Value	Reference range
Histoplasma antigen	<0.2	<0.2mg/mL
Urine drug screen	Negative	Negative

**Figure 2:**
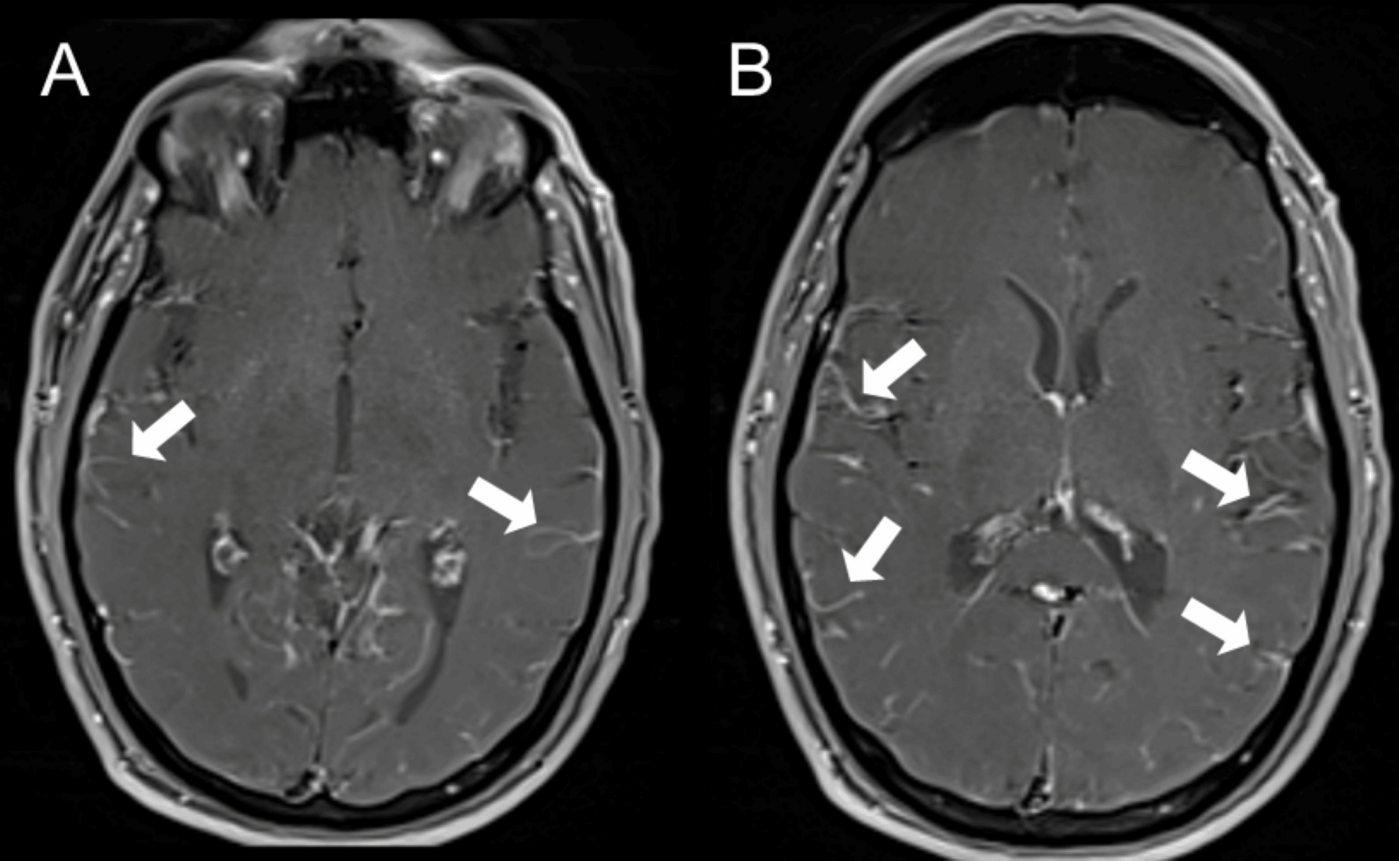
Contrast-enhanced axial T1-weighted sequence shows diffuse leptomeningeal enhancement (A and B). The enhancement is both pia-arachnoid, which extends into the subarachnoid spaces of the sulci (arrow).

**Figure 3:**
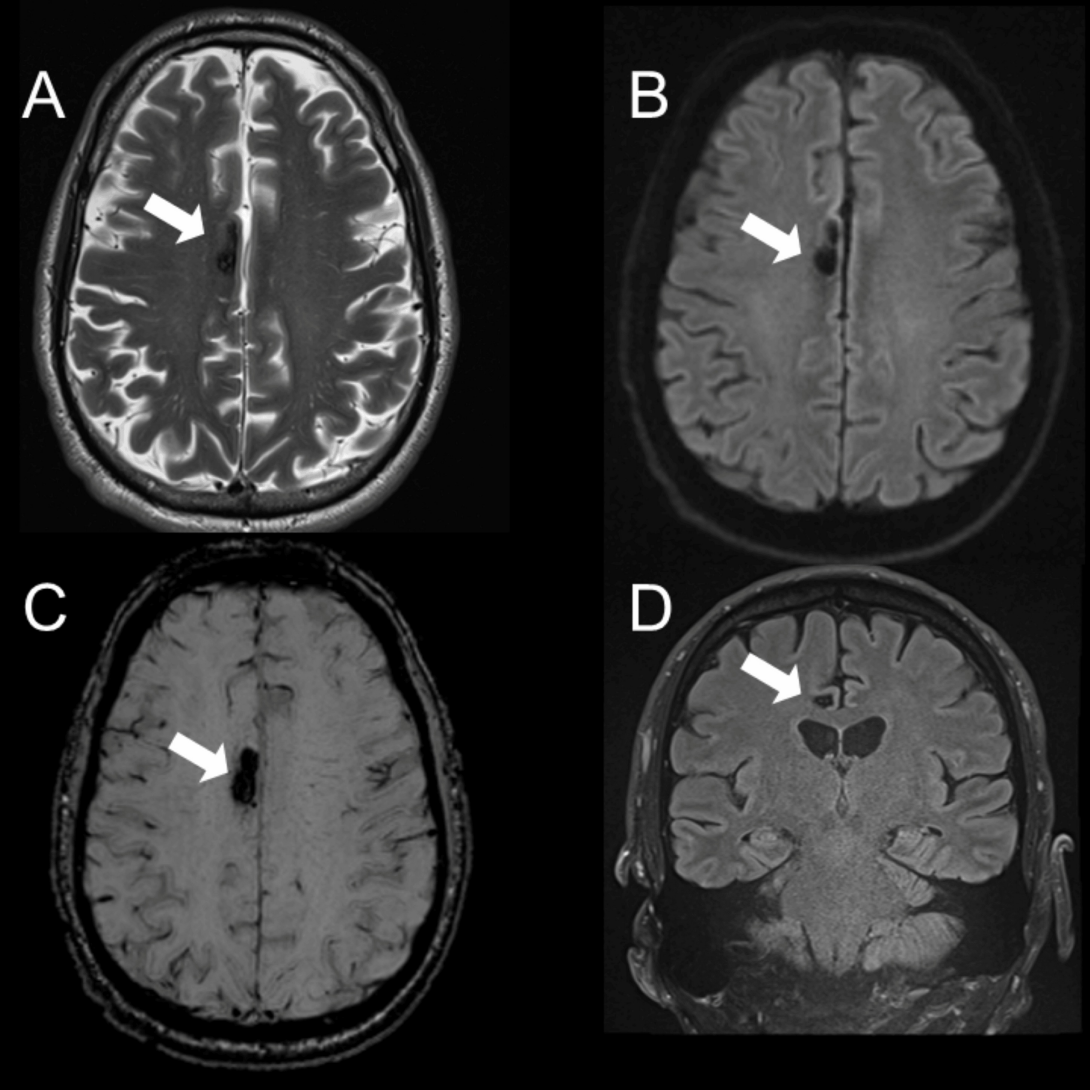
Brain MRI images T2-weighted (A), FLAIR (B, D), and SWI (C) reveal a right frontal cerebral cavernous malformation located just above the corpus callosum (marked by arrow). The lesion exhibits high signal intensity on T2 and FLAIR (A and B) with a surrounding rim of low signal intensity. Additionally, SWI (C) displays a blooming artifact characteristic of cavernoma. FLAIR: fluid-attenuated inversion recovery; SWI: susceptibility-weighted imaging

Brain MRI, with and without contrast, revealed leptomeningeal enhancement concerning for leptomeningitis (Figure 2), as well as an incidental right frontal CCM located just above the corpus callosum (Figure 3).

There was no evidence of hemorrhage or mass effect. Although a positive HHV-6 result on the BioFire meningitis panel may not always indicate active infection, the CSF profile characterized by lymphocytic pleocytosis and significantly elevated protein, together with the MRI findings of leptomeningitis, supports HHV-6 as the most probable etiologic agent for his clinical presentation.

Initially, the patient was started on empiric acyclovir and levetiracetam. After HHV-6 was identified on the BioFire meningitis panel and all other infectious studies returned negative, intravenous ganciclovir (410 mg twice daily) was initiated for one week, followed by oral valganciclovir (900 mg twice daily). Levetiracetam was continued at a dose of 1.5 g twice daily. The patient was extubated on hospital day 4. By day 7, his cognitive function had returned to baseline, and he was discharged home on oral antivirals for an additional week, along with the continuation of anti-seizure medication and outpatient follow-up.

## Discussion

HHV-6 meningoencephalitis is typically associated with immunocompromised hosts; its occurrence in immunocompetent individuals is rare [1]. In this case, the BioFire meningitis panel detected HHV-6 in an immunocompetent patient. Although a positive HHV-6 result can reflect latent or reactivated infection [6] and is therefore often considered a diagnosis of exclusion, several findings support active HHV-6 infection in this case. The CSF demonstrated lymphocytic pleocytosis with elevated protein, and a brain MRI revealed leptomeningeal enhancement indicative of leptomeningitis. HHV-6 was the only pathogen detected. The BioFire meningitis panel, which has a reported sensitivity and specificity of approximately 90% and 97%, respectively, tests for multiple pathogens, including *Escherichia coli* K1, *Haemophilus influenzae*, *Listeria monocytogenes*, *Neisseria meningitidis*, *Streptococcus agalactiae*, *Streptococcus pneumoniae*, CMV, *Cryptococcus neoformans*/*Cryptococcus gattii*, enterovirus, HSV-1, HSV-2, human parechovirus, and VZV [5], all of which were negative. Additionally, all other infectious studies, including HIV, syphilis, tuberculosis, *Cryptococcus*, histoplasma, and *Coccidioides*, as well as CSF and blood cultures, were negative. Taken together with the patient’s history of sick contact, these findings strongly suggest HHV-6 as the most likely cause of meningitis. Importantly, the patient’s clinical recovery following treatment with ganciclovir provides strong support for HHV-6 meningitis as the underlying diagnosis.

The cause of this patient’s recurrent episodes of acute encephalopathy, separated by symptom-free intervals, remains uncertain. Notably, he had close contact with a coworker who was diagnosed with aseptic meningitis of unknown etiology three months prior. In the weeks that followed, the patient began experiencing transient neurological symptoms, including speech difficulties, and approximately one month before presentation, he had an episode of acute confusion with unremarkable EEG and MRI findings. These episodes may represent a spectrum of HHV-6 activity, ranging from primary infection to latency and subsequent reactivation. HHV-6 reactivation can trigger CNS inflammation, manifesting as encephalitis or meningitis, and may also include seizures as part of the clinical presentation [2,6]. In this immunocompetent patient, recurrent encephalopathy could reflect HHV-6 reactivation in the context of transient immunologic vulnerability or predisposing structural abnormalities. Recurrent seizures may explain the clinical picture. HHV-6 DNA has been identified in brain tissue from a subset of patients with epilepsy, suggesting a possible link [2]. However, in this patient, EEG revealed generalized encephalopathy without epileptiform discharges, and no seizures were observed clinically. Although the incidental CCM could be epileptogenic, there was no definitive evidence of seizure activity: EEG showed diffuse encephalopathy without epileptiform discharges, and there were no clinical signs of tonic or clonic activity. Nevertheless, levetiracetam was continued at discharge as a precautionary measure.

HHV-6 meningoencephalitis in immunocompetent patients is rarely reported. Aside from a handful of case reports, there are no large-scale reviews [7], and predisposing factors remain poorly characterized. In this case, the patient’s history of remote TBI and the incidental finding of a CCM may offer important clues. While HHV-6 is a ubiquitous childhood virus, its potential to cause CNS infection in immunocompetent adults is under-recognized. Existing case reports have not identified consistent clinical features that distinguish HHV-6 meningoencephalitis from other viral etiologies in immunocompetent hosts [2]. However, emerging evidence suggests that prior brain insults affecting the integrity of the BBB may facilitate HHV-6 neuroinvasion [1,6]. We proposed that structural vulnerabilities of the CNS, such as CCMs or prior TBI, may predispose patients to viral entry.

CCMs are vascular malformations composed of clusters of dilated, thin-walled capillaries that lack intervening normal brain parenchyma. These lesions are known to cause chronic focal disruption of the BBB, reflected by hemosiderin deposition, gliosis, and perilesional edema due to low-grade inflammation. Studies using dynamic contrast-enhanced MRI and histopathology have demonstrated persistent BBB permeability in and around CCMs, even in the absence of active hemorrhage [3]. Therefore, CCMs may serve as an anatomical “weak point” that facilitates viral neuroinvasion. Therefore, CCMs may serve as an anatomical 'weak point' that facilitates viral neuroinvasion. For example, a recent case report describing the co-occurrence of familial cerebral cavernous malformations and tuberculous meningitis may support this hypothesis [8].

TBI is another well-documented cause of BBB disruption [4,9,10]. Mechanical injury to cerebral endothelial cells during head trauma impairs tight junction integrity, leading to increased BBB permeability [9]. This dysfunction may persist long after the acute event, potentially allowing peripheral immune cells or latent neurotropic viruses such as HHV-6 to gain access to the CNS [2]. As HHV-6 establishes lifelong latency in mononuclear cells, the development of meningitis/encephalitis typically requires some degree of immune dysregulation or BBB compromise [11]. In immunocompetent individuals, a history of head trauma may represent such a facilitating factor, permitting viral reactivation or entry into the CNS.

Our patient’s incidental CCM and history of remote TBI may have independently or synergistically facilitated HHV-6 neuroinvasion. Although a direct causal link cannot be definitively established, the possible anatomical vulnerabilities identified in this case highlight the need to consider structural risk factors when HHV-6 meningitis/encephalitis occurs in immunocompetent individuals. Predisposing factors such as prior trauma or the presence of a CCM warrant further investigation in cases of HHV-6 meningoencephalitis among immunocompetent patients. Once such factors are recognized, HHV-6 should be considered a serious etiologic possibility, rather than merely a diagnosis of exclusion, in the evaluation of viral meningoencephalitis.

## Conclusions

This case highlights a rare yet important presentation of HHV-6 meningitis in an immunocompetent adult without overt immunosuppressive risk factors. The coexistence of a CCM and a remote history of TBI suggests that focal disruption of the BBB may facilitate viral entry into the CNS. Clinicians should maintain a high index of suspicion for HHV-6 in the differential diagnosis of acute encephalopathy, even in immunocompetent patients, particularly when neuroimaging reveals structural brain lesions or there is a history of prior head trauma. Early recognition and antiviral treatment may improve clinical outcomes. Further research is needed to elucidate the predisposing factors and the pathophysiological mechanisms linking BBB dysfunction to HHV-6 neuroinvasion in immunocompetent individuals.
